# Patterns of Acute Poisoning in Childhood in Zagazig, Egypt: An Epidemiological Study

**DOI:** 10.1155/2014/245279

**Published:** 2014-10-29

**Authors:** Basheir A. Hassan, Mohamed G. Siam

**Affiliations:** ^1^Pediatrics Department, Faculty of Medicine, Zagazig University, Zagazig 44519, Egypt; ^2^Forensic Medicine and Clinical Toxicology Department, Faculty of Medicine, Zagazig University, Zagazig 44519, Egypt

## Abstract

*Background*. Acute poisoning represents one of the most common medical emergencies in childhood. In view of paucity of literature on accidental poisoning among children in Egypt, this study was designed to describe the pattern of childhood poisoning in Zagazig University Hospitals. *Patients and Methods*. This retrospective study included 300 children up to 12 years with acute poisoning admitted to the Pediatric Department and Poisoning Treatment Unit, Zagazig University Hospitals, from January 2011 to August 2012. Complete epidemiological and clinical data were recorded and analyzed. *Results*. Three hundred of poisoned children were enrolled in this study. Children from 1 to 6 years were more liable to poisoning (81%). More boys than girls were poisoned at all age groups. The majority of all cases (99%) were due to accidental poisoning. Overall, 32% of the poisoned cases were living in Zagazig city while 68% were living in the rural areas. The presenting symptoms were classic in 60% of the cases. Pesticides, therapeutic drugs, and cleaning and disinfectant agents were the most frequent poisoning agents (28.7%, 22.7%, and 17.0%, resp.). In 86.0% of cases, observation with or without supportive measures together with decontamination and specific antidote therapy whenever needed was sufficient. *Conclusion*. Most of the poisonings were due to accidental ingestions by infants and young children. Pesticides and medications were the most commonly involved agents.

## 1. Introduction

Acute poisoning is a common cause of morbidity and mortality among children accounting for more than 1 million cases annually reported to the Toxic Exposure Surveillance System (TESS) of the American Association of Poison Control Centers (AAPCC) [1, 2].

Poisoning is also the third most common emergencies of pediatrics leading to high social and economic burden [1]. The high prevalence of acute poisoning in children is attributed to the curiosity of the children especially those aged less than 5 years to virtually taste or swallow harmful substances [2].

The prevalence and types of poisoning vary considerably across the world and depend on socioeconomic status and cultural practices, as well as on local industrial and agricultural activities [3].

In view of the paucity of the literature on accidental poisoning among children in Egypt, this retrospective study was designed to describe the pattern of childhood poisoning at the Poisoning Treatment Unit, Faculty of Medicine, Zagazig University Hospitals, to compare the results with studies from other countries, and to help in preventing accidental poisoning in the future.

## 2. Patients and Methods

This observational study was carried out in Zagazig University Hospitals during the period from January 2011 to August 2012. The study population consisted of children up to 12 years of age who were referred to the Pediatric Emergency Department with acute poisoning and admitted to the general pediatric ward, the poisoning treatment unit, or the pediatric intensive care unit (PICU). Poisoned children presented with or suspected to have coma, respiratory distress, cardiovascular instability (severe hypotension or cardiac arrhythmias), or any organ failure were managed in ICU.

Exclusion criteria included cases presented with allergic reactions to food or poisoning stemming from infectious agents, cases of chronic poisoning due to lead exposure, or with other effects of repeated or prolonged exposure to toxic agents, cases associated with work place hazards, cases of drug or chemical abuse, and cases who were not admitted to our hospital.

We reviewed the medical records and collected data of all cases. These records were consistently assessed regarding age, gender, agents involved in the exposure, exposure time, type, route, reason, presenting symptoms, and signs especially these consistent with toxidromes, management, clinical course, laboratory investigations, and final outcome.

Zagazig is a town in eastern part of the Nile Delta and the province of Sharkia Governorate. Zagazig includes agricultural, industrial, business, and residential regions. According to CAPMAS 2006 [4], Sharkia Governorate has a total population around five millions; 23% of the populations live in urban areas while 67% live in rural areas.

We used statistical descriptive methods (frequency and percentage) to analyze data. The study protocol was approved by the Pediatric Ethical Committee in Zagazig University.

## 3. Results

The total number of poisoned children that could be enrolled during this period was 300; the patients were divided into 4 groups according to their ages: younger than 1 year, those between 1 and 3, those between 4 and 6 years, and those between 7 and 12 years.

Almost 2% of the cases involved children below 1 year, with 66.6% males and 33.3% females. The majority of cases (81%) were between 1 and 6 years with 55.5% males and 44.4% females. Children between 7 and 12 years composed 17% of the cases with 58.8% males and 41.2% females. In all age groups more male cases (56.3%) were found as compared to female cases (43.6%).

Thirty-two percent of the poisoned cases were living in Zagazig city while the majority of cases (68%) were living in rural areas. In 91% of cases the accident happened at home while in 9% of cases the accident occurred outside the home.

Nearly all the cases of poisoning (99%) during this period were accidental while the remaining cases are intentional. In 91% of the cases, the cause of poisoning was a single agent, while in 9% of the cases more than one toxic agent was involved. In 72% of the cases the oral route was the route of poisoning while in 28% of the cases other routes were incriminated. Other routes include inhalation and dermal contact in the form of bite or sting.

Table 1 summarizes all these demographic and clinical data.

The presenting symptoms were classic in 70% of the cases while in 30% of the cases the clinical course did not coincide with any constellation of symptoms or no symptoms appeared at all.

**Figure 1:**
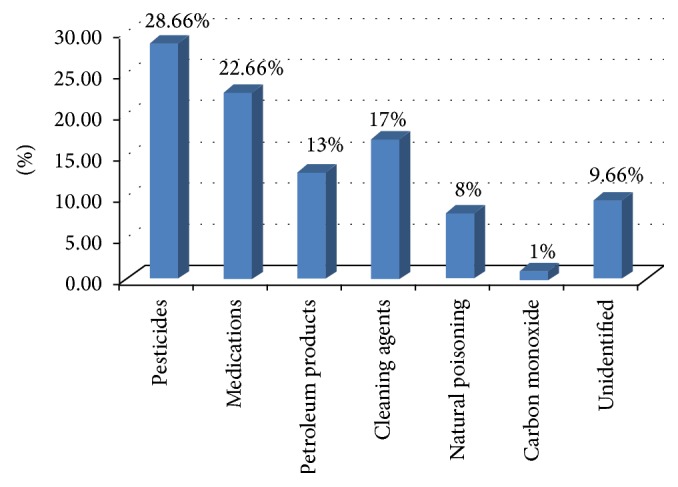
Distribution of major substances involved in childhood poisonings.

The distribution of major substances involved in poisoning of children was presented in Figure 1.

In most of cases, pesticide poisoning was 28.7% of the total cases. Pesticides include organophosphorus insecticides (38.4%), Carbamates (36.1%), and rodenticides (22.1%) in the form of zinc phosphide and long acting anticoagulants. The pesticide used was not identified in 3.5% of the cases.

Therapeutic drugs constituted 22.7% of the causes of poisoning. Of these agents poisoning with neurological medications was 29.4% of the cases and analgesics and nonsteroidal anti-inflammatory drugs (NSAIDs) were implicated in 22.1% of the cases. Over-the-counter (OTC) drugs in the form of cold medications and cough suppressants were found to be the cause of poisoning in 19.1% of the cases while cardiovascular drugs were 13.2% of the cases of poisoning.

Cleaning and disinfectant agents were involved in 17.0% of the cases; the most commonly involved agents were bleaches like sodium hypochlorite (Clorox), disinfectant, like chloroxylenol (Dettol), laundry detergents, phenol (carbolic acid), and potash.

Petroleum products in the form of kerosene, benzene, and others were implicated in 13.0% of the cases.

Carbon monoxide (CO) poisoning was the cause of poisoning in 1.0% of the cases. Animal bites/stings in the form of snake bite and scorpion stings involved 8.0% of the cases, while the unidentified causes were attributed to 9.7% of the cases.

Thirty-one (10.3%) patients were admitted to the intensive care unit (ICU) and 3.7% were discharged against medical advice (DAMA) after taking the consent of legal guardian, while in the majority of cases (86%), observation with or without supportive measures together with decontamination and antidote therapy whenever needed was sufficient.

## 4. Discussion

Acute poisoning represents one of the most common medical emergencies in childhood. Determination of epidemiological characters of childhood poisoning is of paramount importance for preventive measures and treatment plane. In the present study children belonging to age group of 1–6 years represented the largest proportion (81%). This result was in agreement with Lamireau et al. study [5]. Also several studies in Turkey reported that 51–73% of all poisoning cases were observed in children of 5 years of age [6–9]. Poisoning is mostly observed in children belonging to this age group, since they are active and curious and have the tendency to put everything in their mouths [8].

Our study showed a male predominance in all age groups which was in agreement with Lin et al. study, as in some cultures, girls are expected not to engage in outdoor activities or to adopt risk-taking behavior [10].

Our study showed that the number of poisoned children was higher in rural areas than that of urban areas; these results were in agreement with that of O'Connor study, in which children poisoning admission rates have been consistently higher in rural areas than urban areas [11]. This was probably due to the fact that physicians in the rural areas may err on the side of caution and refer children to hospitals even when this was unnecessary; also in urban areas many cases are treated in private hospitals.

In our study the cause of poisoning was mainly accidental, which was in agreement with Agran et al. who reported that poisoning of children up to the age of 10 years tends to be unintentional [12]. In our study, only 3 (1.0%) of the cases were probably intentional, two boys and a girl both were about 12 years old; these results are consistent with other results from Islamic countries; the low incidence of suicide could be associated with the strict forbiddance of suicide by Islam and the religious practice of the majority of the populations [9].

This study showed that 9% of the cases were exposed to more than one agent which was in agreement with the results of Lee et al. [13], while the majority of cases, 91%, ingested a single substance which was in agreement with the study of Hon et al. [14].

The oral route was documented in the majority of poisoned cases, which was similar to the results of Hyder et al. [15].

Pesticides were the most common agent implicated in our poisoned patients which was in agreement with the study of Akhtar et al. [16]. This could be explained by the fact that Zagazig area is one of the major agricultural areas in Egypt, and the easy access and inappropriate usage of pesticides are common.

Poisoning with medications was the second most prevalent agent. These results were in agreement with other studies held in developing countries such as Serilanka [17], Nepal [18], and Ethiopia [8] but not in agreement with recent studies in Taiwan [13], other Asian countries, some western countries, and Turkey [9].

Neurological medications were the predominant agents. Nonsteroidal anti-inflammatory drugs and analgesics were the second cause of poisoning in this group. Analgesics are the predominant agents in USA, Turkey, Malaysia, and Oman [19]. Over-the-counter (OTC) medications accounted for the third most common drugs implicated in poisoning. These findings were similar to the Australian findings [20] but different from those of Litovitz et al. study in USA who found that cosmetics and personal care products, cleaning substances, and plants were the most commonly implicated substances [21].

In the present study, cleaning and disinfectant agents were 17% of the cases of poisoning which was in agreement with Hyder et al. [15] who stated that in Bangladesh, Colombia, Egypt, and Pakistan cleaning agents accounted for 20% of the cases of poisoning.

Petroleum products were attributed to 13% of the cases of poisoning which was in agreement with other studies held in developing countries [16].

The small number of carbon monoxide (CO) poisoning cases in this study was in agreement with the study of Mutlu et al. [9], who reported that in low-income countries, indoor cooking fires in homes with poor ventilation can lead to CO production. The clinical presentation of CO poisoning may be various and nonspecific. Mild clinical signs and symptoms associated with CO poisoning are headache, dizziness, weakness, lethargy, and myalgia; however, severe signs and symptoms such as blurred vision, syncope, convulsion, coma, cardiopulmonary arrest, and death can also accompany CO poisoning [22].

In our study the unidentified poisonous agents were in 9.7% of cases which were in agreement with Holder et al. who stated that sometimes poisoning is correctly diagnosed but the healthcare workers may not recognize the toxic agent that is responsible [23].

In our study most of the patients were symptomatic at admission, with the commonest symptoms being vomiting, drowsiness, and confusion; these results were not in agreement with that of Wiseman et al. [24].

Regarding the outcome, the majority of our patients (86%) completed the treatment period and were discharged properly. Discharge against medical advice (DAMA) was done for 3.7% of patients while thirty one (10.3%) patients were admitted to the intensive care unit.

Potential limitations of this study were its retrospective design and difficulties in data retrieval from old unorganized data collection system.

## 5. Conclusion

Most of the poisonings were due to accidental ingestions by infants and young children. Pesticides and medications were the most commonly involved agents. Good supportive care is the cornerstone of management for childhood poisoning.

Poisoning can be reduced through the use of effective prevention strategies as removing the poisoning agent from the environment (e.g., removal of poisonous plants and removal of fuel sources such as bottled kerosene), replacing the poisoning agent with one of lower toxicity (e.g., replacing aspirin with paracetamol), enforcement of child-resistant packaging of necessary poisonous agents (e.g., medicines, household chemicals, and other toxins), and reducing toxicity of poisoning agents by packaging in nonlethal concentrations or doses and appropriate public education on safe practices of storing medications and toxic household chemicals.

The epidemiological aspects of childhood poisonings should be further assessed by prospectively designed multicenter studies throughout our country.

## Figures and Tables

**Table 1:** Demographic and clinical data of poisoned children.

Variable	Number	%
Age (years)		
Less than one year	6	2%
1–3 years	210	70%
4–6 years	33	11%
7–12 years	51	17%
Sex		
Male	169	56.3%
Female	131	43.7%
Residence		
Urban	96	32%
Rural	204	68%
Place of poisoning		
Home	273	91%
Outside home	27	9%
Mode of poisoning		
Accidental	297	99%
Intentional	3	1%
Route of poisoning		
Oral	216	72%
Other routes	84	28%
System involved		
Gastrointestinal	180	60%
Cardiopulmonary	38	12.6%
Neurological	32	10.6%
Others (skin, eye, and nose)	50	16.6%
Outcome for admission		
Discharge	258	86%
Discharge against medical advice (DAMA)	11	3.7%
Admission to the ICU	31	10.3%
